# Cannabidiol, a Strategy in Aging to Improve Redox State and Immunity in Male Rats

**DOI:** 10.3390/ijms252212288

**Published:** 2024-11-15

**Authors:** Mónica De la Fuente, Noelia Joyera, Judith Félix, Estefanía Díaz-Del Cerro, Beatriz Linillos-Pradillo, Lisa Rancan, Jesús A. F. Tresguerres

**Affiliations:** 1 Department of Genetics, Physiology and Microbiology (Animal Physiology Unit), Faculty of Biological Sciences, Complutense University of Madrid, 28040 Madrid, Spain; njoyera@ucm.es (N.J.); estedi01@ucm.es (E.D.-D.C.); 2 Institute of Investigation Hospital 12 Octubre (imas12), 28041 Madrid, Spain; 3 Department of Biochemistry and Molecular Biology, Faculty of Medicine, Complutense University of Madrid, 28040 Madrid, Spain; beatlini@ucm.es (B.L.-P.); lisaranc@ucm.es (L.R.); 4 Department of Physiology, Faculty of Medicine, Complutense University of Madrid, 28040 Madrid, Spain; guerres@ucm.es

**Keywords:** aging, cannabidiol, rats, oxidative stress, immunity, whole blood cells, spleen, thymus

## Abstract

Aging is characterized by oxidative stress and immune function impairment, and is associated with increased morbidity. Cannabidiol (CBD) has anti-oxidant properties, but its role in aging has been scarcely studied. This work aims to test the effect of CBD on the redox state and immunity during aging in rats. In this study, 15-month-old male Long Evans rats received 10 mg/kg b.w/day of CBD in their diet for 10 weeks and were compared with same-age control and 2-month-old rats serving as a young control group, both following a standard diet. After treatment, they were sacrificed, and the spleen, thymus, and total blood cells were collected. Redox parameters such as glutathione reductase and peroxidase activities, reduced (GSH) and oxidized (GSSG) glutathione concentration, GSSG/GSH ratio, and lipid peroxidation were evaluated. Moreover, immune functions (chemotaxis, natural killer activity, and lymphoproliferation) were analyzed in the spleen. Results show that the 15-month-old control rats exhibited increased oxidative stress and immunosenescence compared to the 2-month-old rats. However, the CBD-treated animals showed higher anti-oxidant defenses, lower oxidants in the spleen, thymus, and blood cells, and better immunity in the spleen than the corresponding age-matched controls. Therefore, CBD administration neutralizes oxidative stress and improves immunity, suggesting it is a strategy for achieving healthy aging.

## 1. Introduction

Aging is a natural process characterized by a progressive and general deterioration of organism functions and a lower capacity for maintaining homeostasis (the dynamic balance by which organisms face all internal and external changes that constantly occur, allowing adaptive responses). Since it is accepted that health involves the preservation of homeostasis [1], aging increases the risk of morbidity and mortality. The aging process begins in adulthood and ends with the death of an individual; thus, its duration determines his/her longevity. The average age of the global population is increasing. However, the current problem is that health span has not kept pace with lifespan, and the burden of age-related diseases has become a global challenge. Thus, research in aging has become a global challenge, especially in finding strategies to slow down aging and maintain health. In this context, having the homeostatic systems (nervous, endocrine, and immune systems) in the best conditions, as well as ensuring their bidirectional connectivity, is an important task. In addition, the oxidation–inflammation theory of aging [2], which integrates the free radical or oxidation theory and the idea of inflamm-aging, proposes that during aging, a state of chronic oxidative and inflammatory stress (excess of oxidant and inflammatory compounds facing the anti-oxidant and anti-inflammatory defenses) develops, which leads to the damage of cell components, including proteins, lipids, and DNA, contributing to the age-related decline of all physiological functions, especially those of the homeostatic systems. Moreover, the immune system, which has been proposed as the best marker of health due to its capacity for producing oxidant and inflammatory compounds (oxidation and inflammation are two very related processes) to carry out its defensive functions, if not well-controlled, seems to be involved in oxi-inflamm-aging and thus in the process of aging [1,3]. Therefore, the possibility of finding compounds that might control these stresses and could improve immunity is, of course, a relevant challenge.

Cannabidiol (CBD) and 9-tetrahydrocannabinol (THC) are the two main cannabinoids of the more than 120 phytocannabinoids identified in *Cannabis*, a genus within the *Cannabaceae* family used by humans for medical purposes for thousands of years. Although CBD and THC possess similar chemical structures, they show different physiological properties. In general, THC is considered to be the primary psychoactive compound in *Cannabis* and influences human somatic, perceptual, and cognitive functions, whereas CBD exhibits a broader therapeutic index and wider pharmacological profile [4,5]. Several studies show that CBD possesses essential properties such as anti-inflammatory and anti-oxidant effects and, consequently, is useful for immunity (thus for cancer, autoimmune diseases, and infections), neuroprotection (neurodegeneration and anxiety), and cardiovascular health, among others [6,7,8,9]. In fact, CBD can act as a powerful anti-oxidant by directly scavenging reactive oxygen species (ROS) such as H_2_O_2_ and chelating transition metals necessary for Fenton reactions, which are behind the non-enzymatic production of ROS [10,11]. However, the pro-oxidant capacity of CBD has also been reported, which depends on the dose used, duration of treatment, and underlying pathology [12].

Although some recent studies have suggested that CBD has protective effects against aging and age-related diseases [13], research on the anti-aging effect of CBD is still in the early stages. In particular, its effects on oxidative stress and immunity in aging animals are scarcely known. In a recent study, it was observed that the administration of 10 mg CBD/kg daily for 10 weeks to adult male rats decreased the amounts of inflammatory and oxidative mediators in the lungs and especially in the liver of 15-month-old animals [14]. In the present work, the effects of that treatment on several parameters of the redox state in the spleen, thymus, and total blood cells, as well as on immune functions in the spleen, were analyzed.

## 2. Results

### 2.1. Oxidative Stress

**Figure 1:**
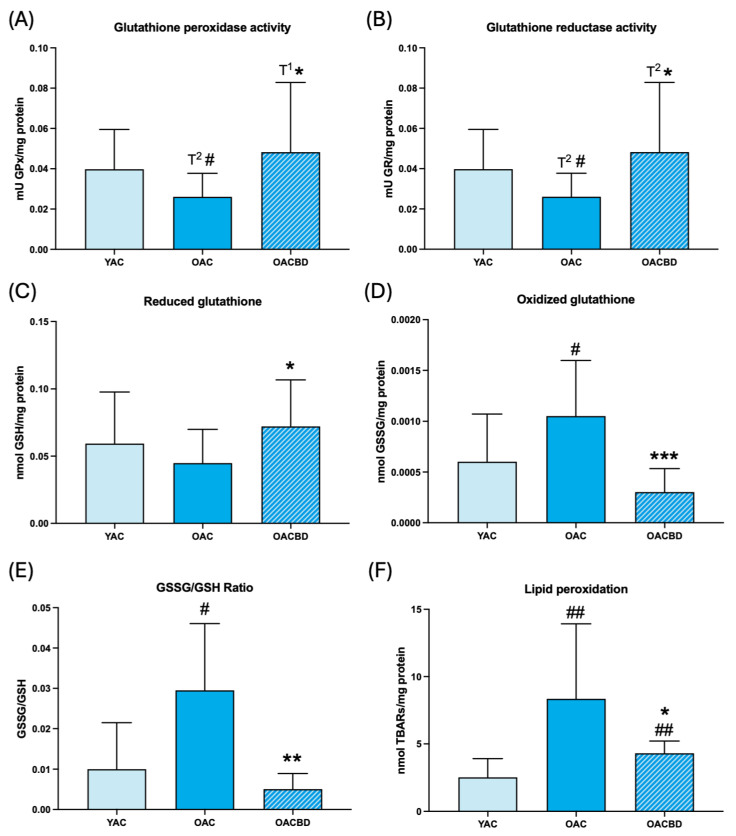
Oxidative stress parameters in total blood cells in young adult control (YAC) (2 months), old adult control (OAC) (15 months), and old adult CBD (OACBD) (15 months). (**A**) Enzymatic activity of glutathione peroxidase (GPx) in mU GPx/mg protein. (**B**) Enzymatic glutathione reductase (GR) activity in mU GR/mg protein. (**C**) Reduced glutathione (GSH) concentration in nmol GSH/mg protein. (**D**) Oxidized glutathione (GSSG) concentration in nmol GSSG/mg protein. (**E**) GSSG/GSH ratio. (**F**) Lipid peroxidation in nmol TBARs/mg protein. Each column represents the mean ± standard deviation (SD) of the values corresponding to the number of animals used in each experimental group (YAC, N = 11; OAC, N = 15; OACBD, N = 14). * *p* < 0.05; ** *p* < 0.01; *** *p* < 0.001; T^1^* *p* = 0.057; T^2^* *p* = 0.058 with respect to the OAC group. # *p* < 0.05; ## *p* < 0.01; T^2^# *p* = 0.058 with respect to the YAC group.

**Figure 2:**
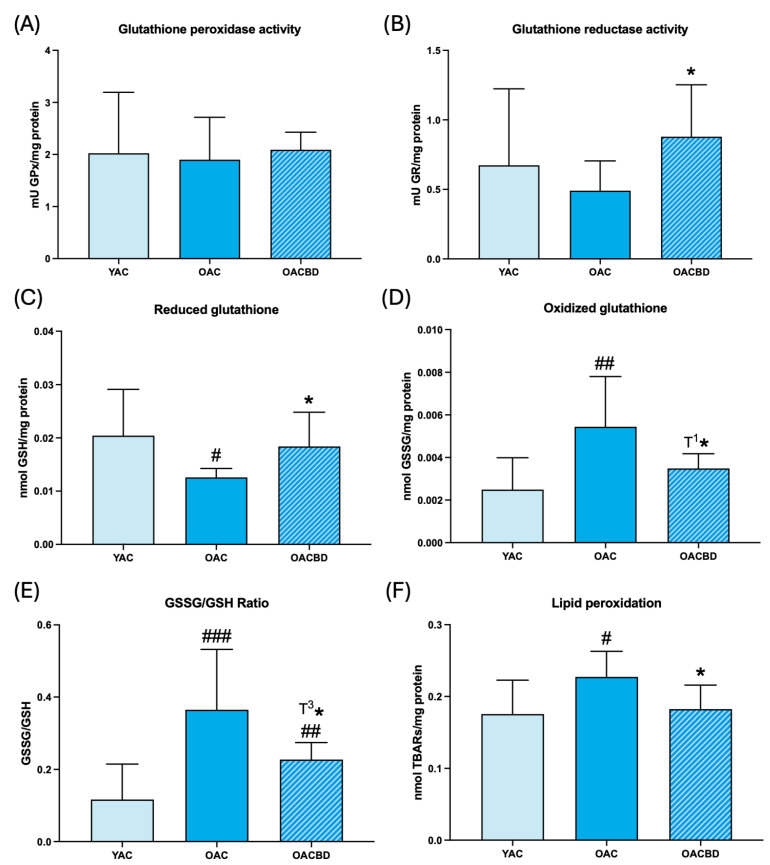
Oxidative stress parameters in the spleen in young adult control (YAC) (2 months), old adult control (OAC) (15 months), and old adult CBD (OACBD) (15 months): (**A**) Enzymatic activity of glutathione peroxidase (GPx) in mU GPx/mg protein. (**B**) Enzymatic glutathione reductase (GR) activity in mU GR/mg protein. (**C**) Reduced glutathione (GSH) concentration in nmol GSH/mg protein. (**D**) Oxidized glutathione (GSSG) concentration in nmol GSSG/mg protein. (**E**) GSSG/GSH ratio. (**F**) Lipid peroxidation in nmol TBARs/mg protein. Each column represents the mean ± standard deviation (SD) of the values corresponding to the number of animals used in each experimental group (YAC, N = 11; OAC, N = 15; OACBD, N = 14). * *p* < 0.05; T^1^* *p* = 0.057; T^3^* *p* = 0.059 with respect to the OAD group. # *p* < 0.05; ## *p* < 0.01; ### *p* < 0.001 with respect to the YAC group.

The results of oxidative stress parameters are shown in Figure 1 (blood cells) and Figure 2 (spleen), as well as in Table 1 (thymus). When the values obtained in 15-month-old (old adult control: OAC) rats are compared with those in 2-month-old (young adult control: YAC) animals, it can be observed that, in general, the anti-oxidant defenses studied showed lower values in the first group than in the second one. Thus, in blood cells, the GPx (Figure 1A; *p* = 0.057) and GR (Figure 1B; *p* = 0.057) activities were lower in OAC than in YAC rats. In the spleen, the concentration of GSH was lower in OAC than in YAC rats (Figure 2C; *p* < 0.05). In the thymus, the three anti-oxidant defenses were lower in OAC animals (Table 1; *p* = 0.058 and *p* = 0.057 for GPx and GR, respectively, and *p* < 0.01 for GSH concentration) than in YAC rats. With respect to the analyzed oxidants, the concentrations of GSSG in OAC animals were higher than in YAC rats in blood cells (Figure 1D; *p* < 0.05) and the spleen (Figure 2D; *p* < 0.001). The GSSG/GSH ratio, a clear marker of oxidative stress [15], was higher in OAC rats than in YAC animals, in the blood cells (Figure 1E; *p* < 0.05), spleen (Figure 2E; *p* < 0.001), and thymus (Table 1; *p* < 0.001). Moreover, lipid oxidative damage, measured as TBAR concentration, was higher in the blood cells (Figure 1F; *p* < 0.01), spleen (Figure 2F; *p* < 0.05), and thymus (Table 1; *p* < 0.05) in OAC rats than in YAC animals.

Considering the effects of a diet supplemented with CBD, we observed that the anti-oxidant defenses showed higher values than the corresponding controls of the same age. Thus, in blood cells, GPx (Figure 1A; *p* = 0.057), GR (Figure 1B; *p* = 0.058), and GSH (Figure 1C; *p* < 0.05); in the spleen, GR and GSH (Figure 2B, C; *p* < 0.05); and in the thymus, GPx (Table 1; *p* < 0.05) and GR (Table 1; *p* < 0.01) showed higher values in old adult rats supplemented with CBD (OACBD) compared to their corresponding controls (OACs), and were similar to those in YAC animals or even higher, as seen in the GR activity of the thymus (Table 1; *p* < 0.05). With respect to the GSSG concentration and GSSG/GSH ratio, the values in OACBD rats in comparison to the OAC animals were lower in blood cells (Figure 1D,E; *p* < 0.001 and *p* < 0.01, respectively) and the spleen (Figure 2D,E; *p* = 0.057 and *p* = 0.059, respectively), but GSSG concentrations were higher (Table 1; *p* < 0.05) in the thymus. The lipid peroxidation values in OACBD rats were also lower in the blood cells (Figure 1F; *p* < 0.05), spleen (Figure 2F; *p* < 0.05), and thymus (Table 1; *p* < 0.05) in comparison to the corresponding OAC animals. The positive effects of the ingestion of CBD resulted in oxidant and lipid damage values generally similar to those in YAC rats, except for TBAR concentration in blood cells (Figure 1F; *p* < 0.01) and the GSSG/GSH ratio in the spleen (Figure 2E; *p* < 0.01), which remained higher in the OACBD group than in the YAC group.

### 2.2. Immune Function in the Spleen

**Figure 3:**
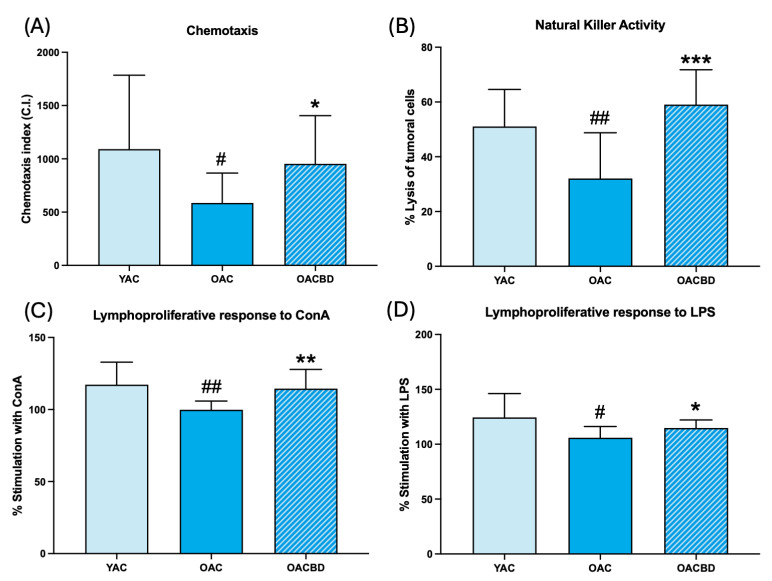
Immune function parameters in spleen leukocytes of young adult control (YAC) (2 months), old adult control (OAC) (15 months), and old adult CBD (OACBD) (15 months): (**A**) Chemotaxis index: number of phagocytes in the filter. (**B**) Natural killer (NK) activity, shown as the percentage of tumoral cell lysis. (**C**) Lymphoproliferative response to concanavalin A (ConA), shown as the percentage of stimulation. (**D**) Lymphoproliferative response to lipopolysaccharide (LPS), shown as the percentage of stimulation. Each column represents the mean ± standard deviation (SD) of the values corresponding to the number of animals used in each experimental group (YAC, N = 11; OAC, N = 15; OACBD, N = 14). * *p* < 0.01; ** *p* < 0.01; *** *p* < 0.001 with respect to the OAC group. # *p* < 0.05; ## *p* < 0.01 with respect to the YAC group.

The results of the immune functions analyzed in cells from the spleen are shown in Figure 3. The OAC rats had lower values of chemotaxis (Figure 3A; *p* < 0.05), NK antitumoral activity (Figure 3B; *p* < 0.01), and proliferative response of lymphocytes to ConA (Figure 3C; *p* < 0.01) and LPS (Figure 3D; *p* < 0.05), in comparison to those of YAC animals. Thus, the OAC group presented characteristics of immunosenescence. The animals supplemented with CBD (OACBD group) showed higher values for these functions than those in OAC animals for chemotaxis (Figure 3A, *p* < 0.05), NK activity (Figure 3B, *p* < 0.001), and lymphoproliferative response to ConA (Figure 3C; *p* < 0.01) and LPS (Figure 3D; *p* < 0.05), with values similar to those in YAC rats.

## 3. Discussion

The results of this study show that treatment with CBD for 10 weeks in 15-month-old rats has beneficial effects by controlling oxidative stress in three locations of the immune system such as blood cells, the thymus, and the spleen, as well as improving the functions of spleen leukocytes. Since improved immunity and redox state are associated with good health maintenance and slowing down aging [16,17], CBD could be considered a good candidate for “anti-aging therapy”, as previously proposed [13].

In the male Long Evans rats used, the presence of oxidative stress (increased oxidative compounds and decreased anti-oxidant defenses) is shown in the blood, spleen, and thymus of 15-month-old control animals (old adult control group; OAC). Thus, in all samples, the GSSG/GSH ratio values were higher in the tissues of OAC rats than in those from the 2-month-old control rats (young adult control group; YAC). This ratio is a clear marker of oxidative stress since it shows the imbalance between the concentrations of an oxidant, such as oxidized glutathione (GSSG), and a relevant anti-oxidant, such as reduced glutathione (GSH) [15]. A consequence of the presence of oxidative stress is the increased oxidation of lipids, especially polyunsaturated fatty acids (PUFAs) found in cell membranes, resulting in lipid peroxides, such as malondialdehyde (MDA), which was measured by TBAR concentrations. These lipid peroxides are highly reactive and can react with other molecules, leading to damage and dysfunction in cells and tissues [18]. The amounts of these peroxides were higher in samples from OAC animals than in YAC animals.

Since in these rats, 2 months old is considered the start of adult age and 15 months old the start of reproductive senescence [19], the oxidative stress observed in these three immune locations proves that 15-month-old animals (OAC) are clearly aging. In fact, an age-related increase in oxidative stress has been observed in the blood cells of humans [17,20] as well as in the spleen and thymus of mice and rats [21,22,23,24]. Moreover, in general, this oxidative stress shown in the spleen is also associated with oxidative stress in other organs of these animals [25] as well as in peritoneal leukocytes [24]. In the case of peritoneal cells, the greater the oxidative stress they show, the lower the lifespan of the animals [17].

In addition, OAC animals have relevant immune functions with values lower than those in YAC rats, showing an immunosenescence at that age, in agreement with previous results obtained in the spleen of rats [26,27,28]. The values in the spleen leukocyte functions were similar to those obtained in Wistar rats, at least in chemotaxis and NK activity, which were analyzed using the same method [26,27,28]. This immunosenescence is associated with oxidative stress, as observed in several works [2,24]. Moreover, in studies carried out in mice, we have shown that these immune functions in the spleen show age-related changes similar to those in the peritoneum [24], and peritoneal immune cells, lower chemotaxis, NK activity, and lymphoproliferation against mitogens are markers of the rate of aging and predictors of lifespan [16,29].

The supplementation with CBD in 15-month-old animals (the OACBD group) seems to neutralize oxidative stress since, in general, anti-oxidant defenses such as the activities of GPx and GR and the concentrations of GSH were higher, while oxidants such as GSSG concentration, GSSG/GSH ratio, and lipid peroxidation, were lower in the blood and spleen than in OAC rats. These differences were more relevant in blood cells, which agrees with our proposal that this sample is the best for analyzing the components of the glutathione cycle [20]. In addition, these differences in the effects of CBD on the activities of GPx and GR, between the spleen and blood cells, with more significant changes in the latter, could be due to the differences observed in the anti-oxidant response to thiol redox between blood and other tissues, such as the spleen, in rats [30]. Moreover, in rats, CBD increases the activity of GR in blood cells [31] and the activity of GPx in the kidney [32], but we have not found a study in which the effects of CBD on the spleen of rats, in this context, have been previously analyzed. However, differences in the effects of CBD on these enzymes of the glutathione cycle in other rat organs have already been observed, showing that the anti-oxidant effects of CBD are more significant (including mRNA expression of GPx and GR) in the liver than in the lungs of rats [14]. Nevertheless, in rats, CBD has widely shown a significant capacity to decrease the effect of oxidative stress, such as lipid peroxidation, thus demonstrating its powerful anti-oxidant effect [31,32,33,34,35].

In the thymus, the GSSG/GSH ratio of supplemented rats (OACBD) was similar to that in OAC animals due to the higher values of GSSG in this organ. This occurs even though CBD increases the activity of the GR enzyme, which transforms GSSG into GSH, more significantly than that of the GPx enzyme, which, by eliminating peroxides, increases the presence of GSSG. It must be taken into account that in the thymus, the concentration of GSSG does not change in OAC rats with respect to YAC animals, while in other organs, the increase in this oxidant due to age is observed. This is possibly due to the characteristics of this organ, which in rats show lower concentrations of GSSG than in the spleen [36], and its age-related involution, with a great amount of infiltrated fat [37]. Moreover, CBD could exert a weaker protective effect on the thymus, similar to its effect on the lung in comparison with the liver, as observed in a previous study in which CBD increased anti-oxidant defenses more significantly in the liver than in the lung [14]. Although the anti-oxidants of the glutathione cycle are the most used in the control of reactive oxygen species (ROS) produced in aging [38], other pathways could be used in organs such as the thymus and lungs. In fact, lipid peroxidation was lower in the thymus of CBD-treated rats compared to their corresponding controls, which shows that other anti-oxidant defenses could be at work in this organ.

In addition, in the spleen, all the values of the immune functions analyzed were higher in OACBD rats than in the corresponding controls (OACs). Although there are studies that support the idea that CBD is immunosuppressive [39], the functions analyzed in the present work, which are associated with health, aging, and longevity [16,29], were improved in the CBD-treated animals. There are no previous studies with CBD on these leukocyte functions, but in other cells, such as glial cells, migration and proliferation have been increased by CBD [8]. In general, findings suggest that CBD is a potent immunomodulatory drug [40,41] since it has manifested immunosuppressive properties, decreasing pro-inflammatory cytokines in the context of sterile inflammation as well as immunoprotective effects during an infection [9,41]. Thus, although CBD seems to show an apparently dual effect with respect to its immune response, we must take into account that enhanced immune cell activity does not have to be associated with increased inflammation, at least with a sterile inflammation (not occurring in response to a harmful antigen). When immune cells have an adequate functional capacity, they are capable of generating pro-inflammatory compounds in response to pathogens, since this inflammation is necessary for destroying them. However, in the absence of dangerous agents, they produce little inflammation. The opposite occurs when there is a deterioration of these immune cells, as is the case during immunosenescence. Thus, there is evidence that as we age, these cells produce more pro-inflammatory compounds during the basal state and less when they have to respond to an infection agent [42,43]. Therefore, CBD acts as an appropriate immunomodulator, with a positive effect on the immune function, enhancing activities that present an age-related decrease such as chemotaxis, NK activity, and lymphoproliferation in response to mitogens, as it has been observed in the present work, and at the same time, CBD reduces inflammation markers (NFkB, IL-1β, and TNF-α) in the lung and more significantly in the liver, as was shown in a previous work with the same experimental design [14].

Furthermore, the effects of CBD on immune cells, increasing or decreasing their functions, vary depending on the concentration of CBD, the time of exposure, the type of immune cells involved, and the age and health state of individuals [44]. Thus, only long-term treatment with CBD, not the acute short-term effects, produces anti-inflammatory effects on the adaptive immune response [45]. Moreover, it is known that oxidation and inflammation are two processes that always occur together, and therefore, anti-inflammatory compounds also show anti-oxidant properties and vice versa [46]. This would explain the anti-oxidant role that CBD manifests in the present experiment. In this context, it would be convenient to consider whether the benefits of CBD are derived from its inherent anti-oxidant activity or through an increase in the body’s anti-oxidant systems. CBD could exert its anti-oxidant role in both ways since it is known that CBD acts as an anti-oxidant at several levels. CBD can have a direct anti-oxidant effect thanks to its hydroxyl group, which can donate electrons to transform oxidants into inert, less harmful molecules that are easier to eliminate. Moreover, CBD can chelate transition metals necessary for Fenton reactions, which are behind the non-enzymatic production of ROS. In addition to interrupting ROS chain reactions, CBD can also act on the redox balance, modifying the amount and activities of anti-oxidant molecules such as GSH concentration and GPx activity [10,12], among others.

With respect to the dose of CBD used, when it is high, it suppresses immune functions, but the effects are positive in animals fed with a supplemented diet with a moderate amount of CBD [7]. A dose of CBD similar to the one used in this study, 10 mg/kg, was the most effective as an antidepressant in old rats [47], and this dose was also used in a study showing a protective effect on age-related inflammation and oxidative stress in the lungs and especially in the livers of male rats [14].

Considering the age of the individuals, another factor that influences the effects of CBD, we chose rats that were 15 months old, that is, approximately halfway through their aging period. This age was appropriate for this type of studies, since they are not old animals. We must keep in mind that aging is a process that begins in adulthood, and it is a different concept from old age. Therefore, to carry out an intervention to improve aging, it should be done before the subjects reach old age. A mature age, such as 15 months of age in rats, when reproductive senescence begins [19], is an age at which the characteristics of aging are clearly shown, and a positive intervention can help by improving the aging process and thus achieving a healthier longevity. The same intervention in already old animals could have shown a more limited effect. In fact, we have verified that lifestyle strategies applied before a very old age are more effective [35,48]. Moreover, very old rats, such as those 24 months old, are animals that have exceeded the average longevity, and in these survivors, the aging characteristics are unclear. In fact, many immune functions and oxidative parameters in long-lived rats and mice show values more similar to those in adults than in old animals [16,17,26].

Several studies have observed that the administration of anti-oxidant compounds to experimental animals during the aging process helps control immunosenescence and the associated oxi-inflamm-aging, allowing these animals to achieve a longer lifespan [35,49]. The results obtained in the present work support the idea that CBD can control immunosenescence and oxidative stress in aging rats, allowing these animals to age better and live longer. Although many studies are still necessary both in animals and especially at the clinical level, and the translation of what has been observed in rodents to humans has its limitations, CBD could be suggested as a possible candidate to slow down aging and consequently achieve healthy longevity.

## 4. Materials and Methods

### 4.1. Animals and Experimental Design

In this study, 40 Long Evans rats (Janvier, Le Genest Saint Isle, France) were allocated into the following experimental groups: 2-month-old adult control rats (YAC; N = 11), 15-month-old adult control rats (OAC; N = 15), and 15-month-old rats receiving CBD supplementation (OACBD; N = 14). Throughout the study, the animals were housed in the Animal Facility at the Faculty of Medicine, Complutense University of Madrid (Registration No. ES-28079-0000086). The study adhered to the standards set by Royal Decree 53/1, issued in February 2023, which outlines the foundational regulations for the protection of animals used in experimental and scientific research.

In terms of diet, all control rats (YAC and OAC) were provided with a standard diet, whereas the CBD-supplemented group (OACBD) received chow enriched with CBD for a duration of two months. To prepare the CBD-enriched chow, pure CBD extract (Phexia, Madrid, Spain) was first used to prepare a CBD stock solution, which was subsequently incorporated into the standard diet to achieve a final concentration of 200 mg of CBD per 100 g of chow. The administered dose was 10 mg/kg of body weight (b.w.) per day. Rat body weights were monitored weekly, and daily food intake volumes were recorded.

At the end of the study, animals were sacrificed by decapitation in the early morning (8:00 a.m.), following the European Community Council Directives (2010/63/EU). The blood, spleen, and thymus were aseptically collected and cleared of any fat. Blood was collected using sodium citrate as an anticoagulant and centrifuged at 1300× *g* for 15 min to separate plasma from whole blood cells. The total blood cells were re-suspended in a RPMI+ medium (Sigma-Aldrich, St. Louis, MO, USA) and aliquoted in 100 μL portions, and then frozen at −80 °C for subsequent redox state analysis. The spleen was bisected, with one portion used to evaluate immune function, while the other half was frozen in liquid nitrogen and stored at −80 °C for redox analysis. The thymus was also frozen in liquid nitrogen and stored at −80 °C for later redox state assessment.

### 4.2. Oxidative Stress Parameters

For these analyses, total blood cell aliquots were centrifuged at 3200× *g* for 30 min, and the resulting supernatants were collected for further evaluation. Tissue samples from the spleen and thymus were processed similarly: each was adjusted to a concentration of 50 mg tissue/mL in the appropriate buffer for each specific assay. For glutathione reductase activity, a phosphate buffer (pH 7.4, 50 mM) was used; for glutathione peroxidase activity, a phosphate buffer with EDTA (pH 7.4, 50 mM, EDTA 6.3 nM) was employed; to measure oxidized and reduced glutathione concentrations, a phosphate buffer with a higher EDTA concentration (pH 8, 50 mM, EDTA 0.1M) was used; and for TBAR concentration assessment, a phosphate buffer with BHT (pH 7.4, 50 mM, BHT 0.1 mM) was used. The tissue samples were then homogenized on ice and centrifuged at 3200× *g* for 20 min, with only the supernatants retained for analysis of the parameters, as previously described [50].

#### 4.2.1. Glutathione Peroxidase (GPx) Activity

Glutathione peroxidase (GPx) activity was measured using a previously established method with slight modifications [51]. Total GPx activity was assessed by tracking the rate at which reduced glutathione (GSH) is oxidized in the presence of GPx, using cumene hydroperoxide as the substrate. In this reaction, NADPH is oxidized concurrently in the presence of excess glutathione reductase (GR). The reaction progress was monitored spectrophotometrically by measuring the decrease in absorbance at 340 nm over a 5 min period, with readings taken every 40 s. To account for non-enzymatic activity, parallel assays of the uncatalyzed reaction were performed to measure the spontaneous reaction rate between cumene hydroperoxide and GSH without the enzyme. Results were expressed as milliunits of enzyme activity per milligram of protein (mU GPx/mg protein).

#### 4.2.2. Glutathione Reductase (GR) Activity

Glutathione reductase (GR) activity was assessed following an established protocol [52]. Total GR activity was measured spectrophotometrically by tracking the oxidation of NADPH, which is utilized by GR to reduce GSSG. The reaction was monitored at 340 nm over a 4 min period, with readings taken every 40 s following an initial delay of 30 s. Results were reported as milliunits of enzyme activity per milligram of protein (mU GR/mg protein).

#### 4.2.3. Concentrations of Oxidized Glutathione (GSSG) and Reduced Glutathione (GSH)

The concentrations of both reduced (GSH) and oxidized (GSSG) glutathione were measured using a fluorometric method initially developed by Hissin and Hilf in 1976 [53], which was subsequently adapted in our laboratory [54] for 96-well plate analysis. This technique relies on the selective reactivity of GSH and GSSG with the fluorescent probe O-phthaldialdehyde (OPT), under optimal pH conditions—pH 8 for GSH and pH 12 for GSSG. This reaction produces a fluorescent complex, measured at an excitation wavelength of 350 nm and an emission wavelength of 420 nm. Results were expressed as nanomoles per milligram of protein (nmol/mg protein), and the GSSG/GSH ratio was calculated by dividing the measured values of GSSG by those of GSH.

#### 4.2.4. Thiobarbituric Acid Reactive Substance (TBAR) Concentration

Lipid peroxidation was assessed by measuring the formation of thiobarbituric acid reactive substances (TBARs) [55], using the commercially available MDA Assay Kit (Biovision, San Francisco, CA, USA). This assay is based on the reaction between thiobarbituric acid (TBA) and lipid peroxidation byproducts, such as malondialdehyde (MDA), whereby one mole of TBA reacts with two moles of MDA to form a colored complex. The resulting compound was quantified through colorimetric analysis at an absorbance of 530 nm. Results were reported as nanomoles of TBARs per milligram of protein (nmol TBARs/mg protein).

#### 4.2.5. Protein Concentration

Protein quantification was performed on the same supernatants obtained from the analysis of the different redox parameters. The bicinchoninic acid (BCA) method, using the BCA kit, was employed for this purpose. This method relies on the reduction of Cu^2^⁺ ions to Cu⁺, which then bind to BCA, forming a colored complex that absorbs light at 562 nm. Protein concentration was expressed as milligrams of protein per milliliter (mg protein/mL).

### 4.3. Immune Function

#### 4.3.1. Isolation of Spleen Leukocytes

For the evaluation of immune function, half of the spleen was minced with scissors and gently forced through a mesh screen (Sigma-Aldrich, St. Louis, MO, USA). Due to the high concentration of erythrocytes in the resulting suspensions, they were centrifuged using a Ficoll-Hypaque (Sigma-Aldrich, St. Louis, MO, USA) gradient with a density of 1.070 g/mL. The cells from the interface were then re-suspended in RPMI 1640 medium containing L-glutamine (PAA) and supplemented with 10% heat-inactivated fetal bovine serum. Leukocyte counts were determined, and the cell concentration was adjusted to 10⁶ cells/mL. Cell viability was assessed using the trypan blue exclusion assay, and only suspensions with greater than 95% viability were utilized.

#### 4.3.2. Chemotaxis Capacity

Chemotaxis was assessed using a modified version of the Boyden chamber technique (Boyden, 1962) [16]. In this procedure, 300 μL of spleen leukocyte suspension, adjusted to 500,000 cells/mL in Hank’s solution, was placed in the upper compartment of the Boyden chamber, separated by a nitrocellulose filter with 3 μm pores. To induce chemotaxis, 400 μL of formylated peptide (N-formyl-methionyl-leucyl-phenylalanine), a known chemoattractant, was added to the lower compartment. The chamber was incubated for 3 h at 37 °C in a 5% CO₂ atmosphere. After incubation, the cells bound to the filter were fixed with a 50% methanol and 75% ethanol solution, followed by staining with azure-eosin blue (GIEMSA, PANREAC, Barcelona, Spain). The number of cells that migrated through the filter, visible on the underside, was counted using optical microscopy, and the chemotactic index (C.I.) was calculated.

#### 4.3.3. Natural Killer Activity

For this, lactate dehydrogenase (LDH) release, indicative of cytolysis of target cells (tumor cells), was measured using an enzymatic colorimetric kit (Cytotox 96 TM, Promega, Promega, Madison, WI, USA; Boehringer Ingelheim, Germany) based on tetrazolium salts. A suspension of spleen leukocytes, adjusted to 10⁶ cells/mL in culture medium, was combined with murine YAC-1 tumor cells in a 10:1 ratio in 96-well U-bottom culture plates. After a 4 h incubation period, LDH release was quantified by adding the enzyme substrate and measuring absorbance at 490 nm. The following formula was used to calculate the NK activity:

$$\mathrm{Lysis}\;\%=\frac{\mathrm{Problem}\;\mathrm{lysis}-\mathrm{Effector}\;\mathrm{cells}\;\mathrm{spontaneous}\;\mathrm{lysis}-\mathrm{Tumor}\;\mathrm{cells}\;\mathrm{spontaneous}\;\mathrm{lysis}}{\mathrm{Tumor}\;\mathrm{cells}\;\mathrm{total}\;\mathrm{lysis}-\mathrm{Tumor}\;\mathrm{cells}\;\mathrm{spontaneous}\;\mathrm{lysis}}\times 100$$

Problem lysis of effector cells refers to the mean absorbance of wells where lysis is observed as a result of the interaction between effector cells (spleen leukocytes) and target cells (YAC-1 tumor cells). Spontaneous lysis of effector cells denotes the lysis occurring due to the natural death of spleen leukocytes during the experiment. Total lysis of target cells represents the mean absorbance of wells where all tumor cells have been lysed by the addition of a lysis solution. Lastly, spontaneous lysis of tumor cells refers to the mean absorbance attributed to the natural death of the tumor cells throughout the procedure [16].

#### 4.3.4. Lymphoproliferation

Lymphocyte proliferation, both under basal conditions and in response to the mitogens Concanavalin A (ConA) and Lipopolysaccharide (LPS), was evaluated using a commercial BrdU (5-bromo-2-deoxyuridine) cell proliferation ELISA kit (Roche Applied Science, Barcelona, Spain). This assay measures the incorporation of BrdU, a thymidine analog, into the DNA of proliferating lymphocytes. For this analysis, 200 μL of spleen leukocyte suspensions, adjusted to 10^6^ lymphocytes/mL in an RPMI medium supplemented with gentamicin (1 mg/mL) and 10% heat-inactivated fetal bovine serum (Gibco, Billings, MT, USA), were added to sterile 96-well plates. To evaluate basal proliferation, 20 μL of RPMI complete medium was added to the wells, while 20 μL of either ConA or LPS (1 μg/mL) was added to assess mitogen-induced proliferation. Following a 48 h incubation, BrdU was added, and the assay protocol was followed to measure its incorporation into the DNA. Results were recorded as absorbance units (AU). Additionally, the percentage of stimulation was calculated by dividing the mitogen-induced lymphoproliferation by the basal lymphoproliferation, and then multiplying it by 100.

### 4.4. Statistical Analysis

Statistical analyses were conducted using GraphPad Prism 10.1.1 (LLC, San Diego, CA, USA). Data are expressed as the mean ± standard deviation (SD). The normality of the data distribution was assessed using the Kolmogorov–Smirnov test, and the homogeneity of variances was evaluated with Levene’s test. To compare the differences between the groups, the independent sample *t*-test was applied, provided the data conformed to a normal distribution. A *p*-value of less than 0.05 was considered statistically significant.

## 5. Conclusions

In conclusion, the results obtained in the present work support the idea that CBD could allow aging rats to age better and live longer by controlling immunosenescence and oxidative stress.

Although many studies are still necessary both in animals and especially at a clinical level, and the translation of what has been observed in rodents to humans has its limitations, CBD could be suggested as a candidate to slow down aging and achieve a healthier longevity.

## Figures and Tables

**Table 1:** Oxidative stress parameters in the thymus in young adult control (YAC) (2 months), old adult control (OAC) (15 months), and old adult CBD (OACBD) (15 months).

	YAC	OAC	OACBD
	(2 Months)	(15 Months)	(15 Months)
**Glutathione peroxidase (GPx)** (mU GPx/mg protein)	0.27 ± 0.16	0.17 ± 0.08 T^2^#	0.26 ± 0.13 *
**Glutathione reductase (GR)** (mU GR/mg protein)	0.68 ± 0.30	0.47 ± 0.24 T^1^#	1.10 ± 0.65 ** #
**Reduced glutathione (GSH)** (nmol GSH/mg protein)	168.1 * 10^−5^ ± 37.6 * 10^−5^	138 * 10^−5^ ± 14.8 * 10^−5^ ##	145.5 * 10^−5^ ± 9.4 * 10^−5^ #
**Oxidized glutathione (GSSG)** (nmol GSSG/mg protein)	84.5 * 10^−5^ ± 38.7 * 10^−5^	88.7 * 10^−5^ ± 28.3 * 10^−5^	113 * 10^−5^ ± 31.3 * 10^−5^ * #
**GSSG/GSH ratio**	0.55 ± 0.22	1.49 ± 0.62 ###	1.82 ± 0.66 ###
**Lipid peroxidation** (nmol TBARs/mg protein)	10.32 ± 6.77	16.08 ± 5.20 #	11.80 ± 2.98 *

Each column represents the mean ± standard deviation (SD) of the values corresponding to the number of animals used in each experimental group (YAC, N = 11; OAC, N = 15; OACBD, N = 14). * *p* < 0.05; ** *p* < 0.01 with respect to the OAC group. # *p* < 0.05; ## *p* < 0.01; ### *p* < 0.001; T^1^# *p* = 0.057; T^2^# *p* = 0.058 with respect to the YAC group.

## Data Availability

The data presented in this study are available on request from the corresponding author upon reasonable request.

## References

[1] López-Otín C., Kroemer G. (2021). Hallmarks of Health. Cell.

[2] De la Fuente M., Miquel J. (2009). An update of the oxidation-inflammation theory of aging: The involvement of the immune system in oxi-inflamm-aging. Curr. Pharm. Des..

[3] Bauer M.E., De la Fuente M. (2016). The role of oxidative and inflammatory stress and persistent viral infections in immunosenescence. Mech. Ageing Dev..

[4] Pintori N., Caria F., De Luca M.A., Miliano C. (2023). THC and CBD: Villain versus Hero? Insights into Adolescent Exposure. Int. J. Mol. Sci..

[5] Martinez Naya N., Kelly J., Corna G., Golino M., Polizio A.H., Abbate A., Toldo S., Mezzaroma E. (2024). An Overview of Cannabidiol as a Multifunctional Drug: Pharmacokinetics and Cellular Effects. Molecules.

[6] Kopustinskiene D.M., Masteikova R., Lazauskas R., Bernatoniene J. (2022). *Cannabis sativa* L. Bioactive Compounds and Their Protective Role in Oxidative Stress and Inflammation. Antioxidants.

[7] Hassan F.U., Liu C., Mehboob M., Bilal R.M., Arain M.A., Siddique F., Chen F., Li Y., Zhang J., Shi P. (2023). Potential of dietary hemp and cannabinoids to modulate immune response to enhance health and performance in animals: Opportunities and challenges. Front. Immunol..

[8] Martini S., Gemma A., Ferrari M., Cosentino M., Marino F. (2023). Effects of Cannabidiol on Innate Immunity: Experimental Evidence and Clinical Relevance. Int. J. Mol. Sci..

[9] Aziz A.I., Nguyen L.C., Oumeslakht L., Bensussan A., Ben Mkaddem S. (2023). Cannabinoids as Immune System Modulators: Cannabidiol Potential Therapeutic Approaches and Limitations. Cannabis Cannabinoid Res..

[10] Atalay S., Jarocka-Karpowicz I., Skrzydlewska E. (2019). Antioxidative and Anti-Inflammatory Properties of Cannabidiol. Antioxidants.

[11] de Almeida D.L., Devi L.A. (2020). Diversity of molecular targets and signaling pathways for CBD. Pharmacol. Res. Perspect..

[12] Pereira S.R., Hackett B., O’Driscoll D.N., Sun M.C., Downer E.J. (2021). Cannabidiol modulation of oxidative stress and signalling. Neuronal Signal..

[13] Ni B., Liu Y., Dai M., Zhao J., Liang Y., Yang X., Han B., Jiang M. (2023). The role of cannabidiol in aging. Biomed. Pharmacother..

[14] Rancan L., Linillos-Pradillo B., Centeno J., Paredes S.D., Vara E., Tresguerres J.A.F. (2023). Protective Actions of Cannabidiol on Aging-Related Inflammation, Oxidative Stress and Apoptosis Alterations in Liver and Lung of Long Evans Rats. Antioxidants.

[15] Kanďár R. (2016). The ratio of oxidized and reduced forms of selected antioxidants as a possible marker of oxidative stress in humans. Biomed. Chromatogr..

[16] Martínez de Toda I., Maté I., Vida C., Cruces J., De la Fuente M. (2016). Immune function parameters as markers of biological age and predictors of longevity. Aging.

[17] Martínez de Toda I., Vida C., Garrido A., De la Fuente M. (2020). Redox Parameters as Markers of the Rate of Aging and Predictors of Life Span. J. Gerontol. A Biol. Sci. Med. Sci..

[18] Halliwell B., Gutteridge J.M. (1984). Free radicals, lipid peroxidation, and cell damage. Lancet.

[19] Sengupta P. (2013). The Laboratory Rat: Relating Its Age with Human’s. Int. J. Prev. Med..

[20] Diaz-Del Cerro E., Martinez de Toda I., Félix J., Baca A., De la Fuente M. (2023). Components of the Glutathione Cycle as Markers of Biological Age: An Approach to Clinical Application in Aging. Antioxidants.

[21] Vida C., Corpas I., De la Fuente M., González E.M. (2011). Age-related changes in xanthine oxidase activity and lipid peroxidation, as well as in the correlation between both parameters, in plasma and several organs from female mice. J. Physiol. Biochem..

[22] Arranz L., Naudí A., De la Fuente M., Pamplona R. (2013). Exceptionally old mice are highly resistant to lipoxidation-derived molecular damage. Age.

[23] Vida C., González E.M., De la Fuente M. (2014). Increase of oxidation and inflammation in nervous and immune systems with aging and anxiety. Curr. Pharm. Des..

[24] Garrido A., Cruces J., Ceprián N., Vara E., de la Fuente M. (2019). Oxidative-Inflammatory Stress in Immune Cells from Adult Mice with Premature Aging. Int. J. Mol. Sci..

[25] Baeza I., Fdez-Tresguerres J., Ariznavarreta C., De la Fuente M. (2010). Effects of growth hormone, melatonin, oestrogens and phytoestrogens on the oxidized glutathione (GSSG)/reduced glutathione (GSH) ratio and lipid peroxidation in aged ovariectomized rats. Biogerontology.

[26] De la Fuente M., Baeza I., Guayerbas N., Puerto M., Castillo C., Salazar V., Ariznavarreta C., JA F.T. (2004). Changes with ageing in several leukocyte functions of male and female rats. Biogerontology.

[27] Baeza I., Alvarado C., Ariznavarreta C., Castillo C., Tresguerres J.A., De la Fuente M. (2008). Effect of growth hormone treatment on lymphocyte functions in old male rats. Neuroimmunomodulation.

[28] Baeza I., Alvarado C., Alvarez P., Salazar V., Castillo C., Ariznavarreta C., Fdez-Tresguerres J., De la Fuente M. (2009). Improvement of leucocyte functions in ovariectomised aged rats after treatment with growth hormone, melatonin, oestrogens or phyto-oestrogens. J. Reprod. Immunol..

[29] Félix J., Martínez de Toda I., Díaz-Del Cerro E., Gil-Agudo F., De la Fuente M. (2024). The immunity and redox clocks in mice, markers of lifespan. Sci. Rep..

[30] Giannerini F., Giustarini D., Lusini L., Rossi R., Di Simplicio P. (2001). Responses of thiols to an oxidant challenge: Differences between blood and tissues in the rat. Chem.-Biol. Interact..

[31] Biernacki M., Brzóska M.M., Markowska A., Gałażyn-Sidorczuk M., Cylwik B., Gęgotek A., Skrzydlewska E. (2021). Oxidative stress and its consequences in the blood of rats irradiated with UV: Protective effect of cannabidiol. Antioxidants.

[32] İlhan İ., Asci H., Ozmen O., Buyukbayram H., Arlıoglu M., Kurtbolat O. (2024). The renoprotective effects of cannabidiol on lipopolysaccharide-induced systemic inflammation model of rats. Naunyn Schmiedebergs Arch. Pharmacol..

[33] Bielawiec P., Harasim-Symbor E., Sztolsztener K., Konstantynowicz-Nowicka K., Chabowski A. (2021). Attenuation of Oxidative Stress and Inflammatory Response by Chronic Cannabidiol Administration Is Associated with Improved n-6/n-3 PUFA Ratio in the White and Red Skeletal Muscle in a Rat Model of High-Fat Diet-Induced Obesity. Nutrients.

[34] Polanska H.H., Petrlakova K., Papouskova B., Hendrych M., Samadian A., Storch J., Babula P., Masarik M., Vacek J. (2023). Safety assessment and redox status in rats after chronic exposure to cannabidiol and cannabigerol. Toxicology.

[35] De la Fuente M., Cruces J., Hernandez O., Ortega E. (2011). Strategies to improve the functions and redox state of the immune system in aged subjects. Curr. Pharm. Des..

[36] Lacombe P., Kraus L., Fay M., Pocidalo J.J. (1985). Lymphocyte glutathione status in relation to their Con A proliferative response. FEBS Lett..

[37] Yang J., Liu J., Liang J., Li F., Wang W., Chen H., Xie X. (2023). Epithelial-mesenchymal transition in age-associated thymic involution: Mechanisms and therapeutic implications. Ageing Res. Rev..

[38] Diaz Vivancos P., Wolff T., Markovic J., Pallardo F.V., Foyer C.H. (2010). A nuclear glutathione cycle within the cell cycle. Biochemical J..

[39] Nichols J.M., Kaplan B.L.F. (2020). Immune Responses Regulated by Cannabidiol. Cannabis Cannabinoid Res..

[40] Peyravian N., Deo S., Daunert S., Jimenez J.J. (2020). Cannabidiol as a Novel Therapeutic for Immune Modulation. Immunotargets Ther..

[41] Hassan Kalantar Neyestanaki M., Gholizadeh O., Hosseini Tabatabaie F., Akbarzadeh S., Yasamineh S., Afkhami H., Sedighi S. (2024). Immunomodulatory effects of cannabinoids against viral infections: A review of its potential use in SARS-CoV2 infection. Virusdisease.

[42] Arranz L., Lord J.M., De la Fuente M. (2010). Preserved ex vivo inflammatory status and cytokine responses in naturally long-lived mice. Age.

[43] Martínez de Toda I., Vida C., De la Fuente M. (2017). An Appropriate Modulation of Lymphoproliferative Response and Cytokine Release as Possible Contributors to Longevity. Int. J. Mol. Sci..

[44] Maggirwar S.B., Khalsa J.H. (2021). The Link between Cannabis Use, Immune System, and Viral Infections. Viruses.

[45] Pénzes Z., Alimohammadi S., Horváth D., Oláh A., Tóth B.I., Bácsi A., Szöllősi A.G. (2023). The dual role of cannabidiol on monocyte-derived dendritic cell differentiation and maturation. Front. Immunol..

[46] De la Fuente M. (2018). Oxidation and Inflammation in the Immune and Nervous Systems, a Link Between Aging and Anxiety.

[47] Hernández-Hernández E., García-Fuster M.J. (2022). Dose-Dependent Antidepressant-Like Effects of Cannabidiol in Aged Rats. Front. Pharmacol..

[48] Arranz L., De Castro N.M., Baeza I., Maté I., Viveros M.P., De la Fuente M. (2010). Environmental enrichment improves age-related immune system impairment: Long-term exposure since adulthood increases life span in mice. Rejuvenation Res..

[49] Martínez de Toda I., Ceprián N., Díaz-Del Cerro E., De la Fuente M. (2021). The Role of Immune Cells in Oxi-Inflamm-Aging. Cells.

[50] Félix J., Díaz-Del Cerro E., De la Fuente M. (2024). Improvement of Immune Function and Redox State in Several Organs of Old and Prematurely Aging Female Mice After a Short Social Interaction with Adults. J. Gerontol. A Biol. Sci. Med. Sci..

[51] Martínez de Toda I., Vida C., Sanz San Miguel L., De la Fuente M. (2019). Function, Oxidative, and Inflammatory Stress Parameters in Immune Cells as Predictive Markers of Lifespan throughout Aging. Oxid. Med. Cell. Longev..

[52] Massey V., Williams C.H. (1965). On the reaction mechanism of yeast glutathione reductase. J. Biol. Chem..

[53] Hissin P.J., Hilf R. (1976). A fluorometric method for determination of oxidized and reduced glutathione in tissues. Anal. Biochem..

[54] Garrido A., Cruces J., Ceprián N., Corpas I., Tresguerres J.A., De la Fuente M. (2019). Social environment improves immune function and redox state in several organs from prematurely aging female mice and increases their lifespan. Biogerontology.

[55] Mihara M., Uchiyama M. (1978). Determination of malonaldehyde precursor in tissues by thiobarbituric acid test. Anal. Biochem..

